# Mutation analysis of *PALB2* in *BRCA1* and *BRCA2*-negative breast and/or ovarian cancer families from Eastern Ontario, Canada

**DOI:** 10.1186/1897-4287-12-19

**Published:** 2014-08-28

**Authors:** Taila Hartley, Luca Cavallone, Nelly Sabbaghian, Rachel Silva-Smith, Nancy Hamel, Olga Aleynikova, Erika Smith, Valerie Hastings, Pedro Pinto, Marc Tischkowitz, Eva Tomiak, William D Foulkes

**Affiliations:** 1 Department of Genetics, Children’s Hospital of Eastern Ontario, 401 Smyth Rd, K1H 8 L1 Ottawa, ON, Canada; 2 Program in Cancer Genetics, Departments of Oncology and Human Genetics, Gerald Bronfman Centre for Clinical Research in Oncology, McGill University, Montreal, QC, Canada; 3 Lady Davis Institute, Segal Cancer Centre, Jewish General Hospital, McGill University, Montreal, QC, Canada; 4 Department of Pathology, McGill University, Montreal, QC, Canada; 5 Department of Genetics, Portuguese Oncology Institute, Porto, Portugal; 6 Department of Medical Genetics, University of Cambridge, Cambridge, UK; 7 Faculty of Medicine, University of Ottawa, Ottawa, ON, Canada; 8 Department of Medical Genetics, McGill University Health Centre, Montreal, QC, Canada

**Keywords:** *PALB2*, Hereditary breast cancer, Pancreatic cancer, Melanoma, *BRCA1* and *BRCA2* mutation-negative

## Abstract

**Background:**

*PALB2* has emerged as a breast cancer susceptibility gene. Mutations in *PALB2* have been identified in almost all breast cancer populations studied to date, but the rarity of these mutations and lack of information regarding their penetrance makes genetic counseling for these families challenging. We studied *BRCA1/2* -negative breast and/or ovarian cancer families to a) assess the contribution of *PALB2* mutations in this series and b) identify clinical, pathological and family history characteristics that might make *PALB2* screening more efficient.

**Methods:**

The coding region of the *PALB2* gene was analyzed in 175 probands with family histories of breast and/or ovarian cancer ascertained from a single Canadian institution in Eastern Ontario.

**Results:**

We identified 2 probands with *PALB2* mutations that are known or strongly considered to be pathogenic and 3 probands with missense mutations that are possibly pathogenic. One of the identified truncating mutations [c.3113G > A (p.Gly1000_Trp1038del – major product)], has been previously described while the other four mutations [c.3507_3508delTC (p.H1170Ffs*19), c.1846G > C (p.D616H), c.3418 T > G (p.W1140G), c.3287A > G (p.N1096S)] have not been previously reported. Loss of heterozygosity was detected in two breast tumors from one c.3507_3508delTC mutation carrier but not in other available tumors from that family or in tumors from carriers of other mutations.

**Conclusions:**

*PALB2* mutation screening identifies a small, but significant number of mutations in *BRCA1/2* -negative breast and/or ovarian cancer families. We show that mutations are more likely to be found in families with three or more breast cancers as well as other *BRCA2*-related cancers. In our cohort, both clearly pathogenic mutations were identified in premenopausal breast cancer cases (2/77, 2.6%). Testing should be preferentially offered to affected women from such families.

## Introduction

Since first being identified as a BRCA2-interacting protein, Partner and Localizer of BRCA2 (PALB2) has been shown to also interact with BRCA1, effectively bridging these two well-known high-risk breast cancer susceptibility genes and aiding to regulate their function in DNA damage response and homologous recombination [1,2]. Not surprisingly, *PALB2* has emerged in the last few years as an important breast cancer susceptibility gene in its own right (reviewed in [3]).

Germline mutations in *PALB2* have been identified worldwide (reviewed in [4]), albeit rarely (1–4% of breast cancer families negative for *BRCA1/BRCA2* mutations), and these mutations are associated with an increased risk of breast cancer that varies from approximately 2.3 to as high as ~6.0 depending on the mutations being studied and the populations under investigation [5-7]. While the degree of breast cancer susceptibility is still unclear, some studies examining recurrent *PALB2* mutations tested in patients unselected for family history have demonstrated a risk and penetrance as high as those arising from *BRCA2* mutations [6,7].

Similar to mutations in *BRCA1* and *BRCA2*, the risks associated with monoallelic mutations in *PALB2* seem to extend beyond breast cancer. To date, the spectrum of malignancies associated with *PALB2* mutations remains unclear, however mutations confer increased risks for pancreatic cancer [8] and possibly ovarian cancer [9].

With the advent and increasing use of multiplex panels that test *PALB2* alongside the *BRCA* genes [10], the greatest barrier for the implementation of *PALB2* analysis into the clinic is no longer its testing efficiency but instead the lack of clear information and recurrence risks with which to counsel patients should a mutation be identified. Determining *PALB2* mutation status is important, however, as it may allow female relatives of mutation positive patients the opportunity to make informed decisions about options to mitigate their elevated risk for disease. Also, new effective targeted therapeutic options are becoming available (PARP inhibitors) that have shown promising results in *in vitro* studies with *PALB2* deficient cells exhibiting a defect in homologous repair [11].

Given that in order to determine clear criteria for genetic testing, we must first identify the likelihood of finding mutations in different populations, here we report our analysis of *PALB2* in 175 breast and ovarian cancer pedigrees from a clinical cohort in Eastern Ontario, Canada, in which *BRCA1* and *BRCA2* testing failed to identify any causal variants.

## Materials and methods

### Cases and case selections

Participants were accrued from May 2009 to July 2012 at the Eastern Ontario Regional Genetics Program at the Children’s Hospital of Eastern Ontario. For this investigation, we selected participants affected with breast or ovarian cancer who had been previously screened for *BRCA1* and *BRCA2* mutations and excluded individuals with pathogenic mutations. Most individuals (169/175) were tested for *BRCA1* and *BRCA2* mutations by denaturing high-performance liquid chromatography, enhanced mismatch mutation analysis or sequencing. In six of eight individuals of Ashkenazi Jewish (AJ) descent, testing was limited to screening for the three common AJ mutations accounting for 98% of mutations in that population. Two individuals of AJ decent underwent full gene analysis, and 163/175 individuals were screened by MLPA for large insertions/deletions. Two separate cohorts of patients were specified: 1) those recruited May 2009 to September 2010, who were affected with breast or ovarian cancer, met Ontario provincial criteria for *BRCA1* and *BRCA2* genetic testing, and had a minimum BRCAPRO score of 0.10; 2) those recruited September 2010 to July 2012, who met select Ontario provincial criteria, were affected with breast or ovarian cancer, and had no BRCAPRO requirement (see Supplemental Materials for detailed list of Ontario Criteria, Additional file 1: Table S1). The criteria for study entry was changed to make sure we did not miss persons with a family history of pancreas cancer that might not otherwise be included on the basis of the BRCAPRO score.

Enrollment of probands, ascertainment of families and *PALB2* gene analysis proceeded identically for each participant. Their respective geneticist or genetic counselor initially identified potential participants, and informed consent was obtained for those meeting criteria and interested in enrolling. In total, 195 patients were originally accrued. Of these, 13 participants showed initial interest yet failed to submit a blood sample, 4 were found to not meet selection criteria following enrollment. Of the 178 participants tested, in three cases the DNA sample was of insufficient quality. Therefore, of the 195 participants originally enrolled, 175 were eligible and able to be tested. Characteristics of the study participants are given in Table 1. Characteristics of breast tumor specimens are given in Additional file 1: Table S2.

**Table 1:** Characteristics of 175 probands with clinical diagnoses of female unilateral breast cancer, female multiple primary breast cancer, male breast cancer or ovarian cancer

	**Female unilateral**		**Female multiple primary**		**Male breast cancer**		**Ovarian cancer**	
**Number of probands:**	126		24		12		13*	
**Age at Diagnosis**	(N)	(%)	(N)	(%)	(N)	(%)	(N)	(%)
*20-29*	7	5.6	0	0	0	0	1	7.7
*30-39*	26	20.6	5	20.8	0	0	1	7.7
*40-49*	44	34.9	13	54.2	3	25.0	5	38.5
*50-59*	30	23.8	6	25.0	2	16.7	0	0
*60-69*	16	12.7	0	0	3	25.0	5	38.5
*70 and over*	3	2.3	0	0	4	33.3	1	7.7
*Mean (years)*	47.1		43.4		60.8		51.3	
*Range (years)*	23-78		31-58		43-76		23-76	
*Mean time from diagnosis (years)*	4.7		12.4		1.8		10.3	
Ethnicity**	(N)	(%)	(N)	(%)	(N)	(%)	(N)	(%)
*British Isles*	47	37.3	8	33.3	6	50.0	5	38.5
*French Canadian*	27	21.4	5	20.8	1	8.3	5	38.5
*Other European*	19	15.1	7	29.2	2	16.6	2	15
*Ashkenazi Jewish*	8	6.3	1	4.2	0	0	0	0
*Asian*	4	3.2	0	0	1	8.3	0	0
*Mixed Ethnicity*	16	12.7	3	12.5	1	8.3	0	0
*Other or unknown*	5	4.0	0	0	1	8.3	1	7.7

*Includes 4 probands affected with both breast and ovarian cancer.

**All self-reported ethnicities from the side of the family eligible for *BRCA1* and *BRCA2* sequencing based on Ontario guidelines.

Of the 13 patients who were diagnosed with ovarian cancer, four of whom were also diagnosed with breast cancer, over half (7/13) of the ovarian cancers were papillary serous or serous morphology. Two probands had ovarian cancers with mucinous morphology. The other 4 participants were affected with one case each of clear cell carcinoma, carcinosarcoma, mixed mucinous and endometrioid cystadenocarcinoma, and borderline papillary serous tumor of low malignant potential.

Of the 24 cases of female multiple primary breast cancer, 16 cases were bilateral metachronous, 5 cases were bilateral synchronous, and 3 were synchronous and in the same breast.

Three hundred and eleven index cases from suspected hereditary breast and ovarian cancer families from Northern Portugal, all Caucasian, were screened for the N1096S variant only. Cases included 256 female breast cancer patients with an average age at diagnosis of 42.6 yrs, 24 male breast cancer cases and 31 index cases with other (or no) cancer.

### Molecular methods

a. ***PALB2*** **sequencing and MLPA**

We screened the 13 coding exons of *PALB2* (NCBI reference sequences NG_007406.1 and NM_024675.3), by high-resolution melting (HRM) analysis using the LightScanner instrument (Idaho Technologies Inc., Utah, USA). The PCR reactions for HRM were performed in 96 well plates from Bio Rad (Ontario, Canada) using the mastermix and the LCGreen Plus from Transition Technologies (Ontario, Canada). Following amplification, the plates were then transferred to the LightScanner instrument and the melting curves were analysed with the software provided by Idaho Technologies. This technique was used as a presequencing selection for amplicons harboring variants. Samples having a different melting curve than the majority were analysed by Sanger sequencing. The primer sequences and the protocol for amplification have been previously published [12]. Samples were also screened for large insertions/deletions overlapping coding regions that would be missed using the PCR-based method above using a commercially available Multiplex Ligation-dependent Probe Amplification mix (MRC-Holland, Amsterdam, Netherlands) using the supplier’s recommended protocol.

b. **c.3287A > G (p.N1096S) screening**

Screening for the c.3287A > G (N1096S) variant in 311 Portuguese index cases was performed using KASPar® SNP genotyping on demand by KBiosciences on a Roche LightCycler® 480 Real-Time PCR System.

c. **LOH testing in tumor samples**

DNA extracted from formalin-fixed, paraffin-embedded (FFPE) tumors that were macro-dissected to enrich for tumor cells was used to perform loss of heterozygosity (LOH) analysis in mutation carriers. The region surrounding each variant was amplified by PCR using variant-specific primers (Additional file 1: Table S3) that were designed using the Primer3 software (http://bioinfo.ut.ee/primer3-0.4.0/). Amplicons were analysed by Sanger sequencing.

d. Expression analysis of c.3507_3508delTC (p.H1170Ffs*19)

Lymphoblastoid cell lines (LCLs) harboring the c.3507_3508delTC (p.H1170Ffs*19) mutation were grown in Dulbecco’s Modified Iscove’s Media (Life Technologies Burlington, Ontario, Canada). RNA was extracted from 10 million cells using Trizol reagent (Life Technologies Burlington, Ontario, Canada) following the manufacturer’s protocol. 1 ug of RNA was used for reverse transcription (Qiagen California, USA) following the manufacture’s protocol, followed by cDNA amplification of *PALB2*. The primers used were: forward primer, 5’-GCAGGCTGGCAGGTTCCT-3’ (covers the junction of exon 12 and 13 of PALB2 in the cDNA); and reverse primer, 5’-CATCCAAGATCAGTGGTGCTA-3’ (in the 3’UTR).

## Results and discussion

Mutation analysis of *PALB2* identified two monoallelic deleterious mutations in 2/126 (1.6%) women with diagnoses of unilateral breast cancer. Of the two mutations, c.3113G > A has been previously reported by several groups and is thought to be a founder mutation from the United Kingdom [5,7,12-15]. It has been shown to produce three distinct transcripts resulting in p.Gly1000_Trp1038del (56%), p.Thr1029Ilefs*2 (40%) and p.Trp1038* (4%) [12] (Additional file 1: Figure S1). The frameshift mutation c.3507_3508delTC (p.H1170Ffs*19) has not been previously reported.

**Figure 1:**
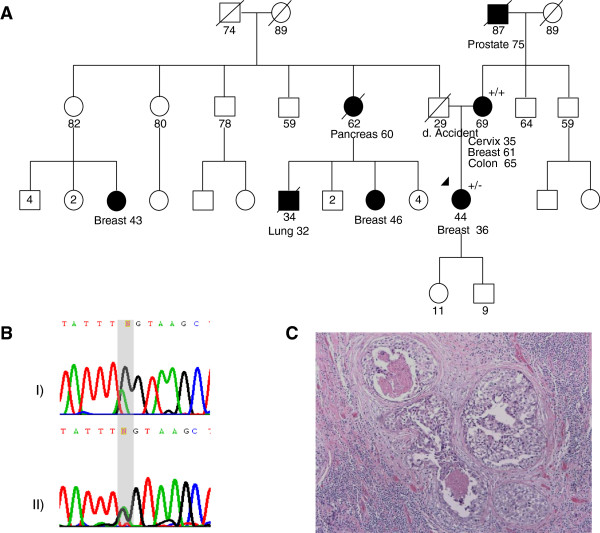
**Pedigree and LOH Testing in Tumors of c.3113G > A Mutation Family. (A)** Pedigree of the family carrying the c.3113G > A mutation. Circles = females; Squares = males. Filled symbols = affected with cancer. Slashed symbols = deceased. +/+ = wild-type; +/- = heterozygous carrier; d. = death, numbers denote age, the arrow denotes the proband. **(B)** Loss of heterozygosity analysis of the proband’s breast cancer shows both alleles are still present in the tumor. **(C)** Hematoxylin-eosin stain of the tumor tissue analyzed in panel **B**.

The index case with the *PALB2* c.3113G > A mutation (Figure 1A) was of British descent and had been diagnosed with invasive ductal (ER+, PR+, HER2-) breast cancer at 36 years of age. Evaluation of her mother revealed that the mutation was most likely paternally inherited. Her paternal family history is significant for an aunt diagnosed with pancreatic cancer at 60 and two first cousins with breast cancer diagnosed in their 40s.

**Figure 2:**
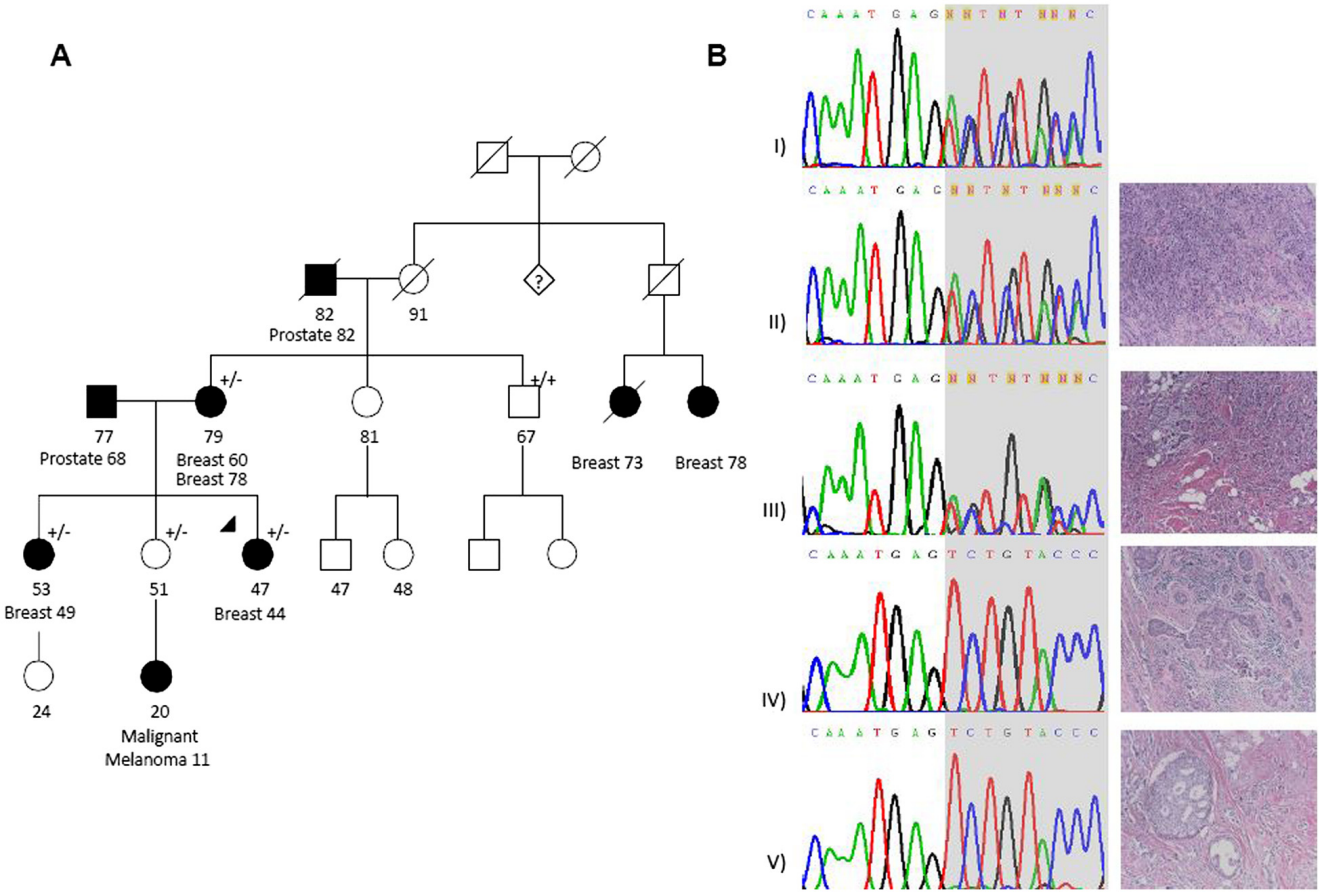
**Pedigree and LOH Testing in Tumors of c.3507_3508delTC Mutation Family. (A)** Pedigree of the family carrying the c.3507_3508delTC mutation. Circles = females; Squares = males. Filled symbols = affected with cancer. Slashed symbols = deceased. +/+ = wild-type; +/- = heterozygous carrier; numbers denote age, the arrow denotes the proband. **(B)** Loss of heterozygosity (LOH) analysis in tumor tissue from mutation carriers. The reverse sequence is shown. A hematoxylin-eosin stain of the tumor tissue analyzed is shown to the right of each chromatogram. I) Normal tissue showing the heterozygous mutation; II) Tumor tissue from the proband, (no LOH); III) Tumor tissue from the proband’s sister (no LOH); IV) Tumor tissue from the left breast of the proband’s mother (loss of the wild-type allele); V) Tumor tissue from the right breast of the proband’s mother (loss of the wild-type allele).

The index case with the *PALB2* c.3507_3508delTC (p.H1170Ffs*19) (Figure 2A) mutation was of British descent and had been diagnosed with invasive ductal (ER+, PR+, HER2+) breast cancer at the age of 44. We evaluated the presence of the mutation in samples from four of the proband’s relatives, from whom genetic material was available; two individuals (the proband’s mother and sister) affected with breast cancer were carriers of the family mutation. The proband’s unaffected sister, who has a daughter with a diagnosis of malignant melanoma at age 11, was also found to carry the familial mutation. An unaffected uncle was found to not carry the familial mutation.

Three additional missense variants predicted to be pathogenic by Sorting Intolerant From Tolerant (SIFT) [16], Polymorphism Phenotyping (Polyphen) [17], MutationTaster [18] and Consensus Deleteriousness (Condel) [19] were identified (Table 2). The first, c.1846G > C (p.D616H) was observed in a proband diagnosed with unilateral breast cancer from a French Canadian family (Additional file 1: Figure S2A). The second, c.3418 T > G (p.W1140G), was observed in a proband diagnosed with unilateral breast cancer from a French Canadian and British family (Additional file 1: Figure S2B). Finally, the third variant, c.3287A > G (p.N1096S), was identified in a proband diagnosed with bilateral breast cancer from a Portuguese family (Additional file 1: Figure S2C). Given the previously reported existence of a Portuguese founder mutation in *BRCA2*[20], we considered the possibility that this novel *PALB2* variant was a founder mutation and subsequently analyzed for its presence in a larger Portuguese series. The c.3287A > G (p.N1096S) variant was not observed in an additional 311 Caucasian index cases primarily from Northern Portugal who were negative for *BRCA1* and *BRCA2* mutations. While these variants are possibly pathogenic functionally, c.1846G > C (p.D616H) does not appear to segregate with the disease, and DNA from additional affected individuals was not available to perform informative segregation analysis of the other two variants. In total, 86 participants, including the 5 with known/predicted pathogenic mutations, were identified to carry a *PALB2* variant (Additional file 1: Table S4).

**Table 2:** **Summary of identified variants and tumor characteristics of the *PALB2 *** **carriers**

			**Tumor characteristics**							**In silico analysis**	
	**Mutation**	**Exon**	**Age**	**Type**	**Stage**	**ER**	**PR**	**Her2**	**Previously reported?**	**SIFT**	**Polyphen**
Truncating	**c.3113G > A**,	10	36	IDC	1	Pos	Pos	Neg	yes		
	p.Gly1000_Trp1038del										
	p.Thr1029Ilefs*2										
	p.W1038*										
	**c.3507_3508delTC**	12	44	IDC	1	Pos	Pos	Pos	no		
	p.H1170Ffs*19										
Missense	**c.1846G > C**	4	25	IDC	1	U	U	U	no	0.02	1.00
	p.D616H										
	**c.3418 T > G**	12	32	IDC	1	Pos	Pos	Pos	no	0.00	1.00
	p.W1140G										
	**c.3287A > G**	10	50/58	DCIS/IDC	0/2	NP/Pos	NP/Pos	NP/U	no	0.00	1.00
	p.N1096S										

ER = estrogen receptor; PR = progesterone receptor; HER2 = human epidermal growth factor receptor 2; IDC = infiltrating ductal carcinoma; DCIS = ductal carcinoma in situ; U = unknown; Pos = positive; Neg = negative; NP = not performed.

We performed loss of heterozygosity (LOH) analysis on available breast tumors from the five families. Tumor tissue was not available for testing for any c.3287A > G (p.N1096S) and c.1846G > C (p.D616H) carriers. LOH was observed in both the left and right breast tumors from the mother carrying the c.3507_3508delTC (p.H1170Ffs*19) mutation, but none of the other tumors showed loss of the wild-type allele [Figures 1B and 2B, data not shown for the tumor from the proband with c.3418 T > G (p.W1140G)]. These results are consistent with previous observations that LOH is an inconsistent feature of *PALB2* tumors, even among tumors carrying identical predisposing mutations [13,14,21,22].

In 175 probands tested, we identified *PALB2* mutations that can alter the length of the PALB2 protein. The first, c.3113G > A, was previously identified in multiple unrelated families from Australia, the United States and the UK and is associated with an estimated 91% (95% CI = 44–100) cumulative risk of breast cancer to age 70 [7,23]. As our family with c.3113G > A is of British descent, our results support the hypothesis that c.3113G > A is a founder mutation in the British population. Most recently, Teo *et al.*[23] identified this particular mutation in 8 of 871 (0.92%) probands from “high-risk” breast and/or ovarian cancer families evaluated in familial cancer clinics in Australia and New Zealand.

The c.3507_3508delTC (p.H1170Ffs*19) mutation is located in the final exon (exon 13) of *PALB2*. Sequencing of RNA from cultured LCLs showed expression of the mutant allele (Additional file 1: Figure S3), indicating that mRNA derived from the mutated allele is not subject to nonsense-mediated decay. The frameshift introduces 18 new amino acids that are not seen in the native protein (Additional file 1: Figure S1B), resulting in a mutant protein of 1187 amino acids, compared to 1186 amino acids in the wild-type protein. The predicted structure of PALB2 includes an amino-terminal coiled-coil structure and a carboxy-terminal WD40-repeats motif that has the characteristics of a seven-bladed beta-propeller, a domain commonly involved in protein-protein interactions. The WD40-repeats domain on amino acids 836–1186 provides a binding site for the N-terminus of BRCA2 [24]. Mutations in this region, even if they do not result in nonsense-mediated decay, may lead to alteration of the structure of WD40-repeats (Additional file 1: Figure S1) [5], and many are known to be pathogenic. Thus, independent of the genetic data we present here (segregation and LOH), there is fairly strong *a priori* evidence that this novel mutation c.3507_3508delTC (p.H1170Ffs*19) we describe here is deleterious, probably as a result of disruption of the BRCA1-PALB2-BRCA2 interaction, resulting in defective homologous repair of double-stranded breaks.

In terms of immunohistochemical features seen in the tumors of our participants with pathogenic or suspected pathogenic *PALB2* mutations, although earlier studies performed in Finnish, French Canadian and other European populations suggested a possible overrepresentation of triple negative (basal-like) tumors in *PALB2*-related breast cancers [4,25,26], our overall results support more recent studies of Australasian breast cancer families that have not found any association between *PALB2* mutations and hormone-receptor negative breast tumors [27,28]. Although the number of probands with multiple breast primaries in our series is small (24 cases) it is interesting that, of the tumors that are available for hormone receptor and HER2 analysis in this specific subgroup of patients, an increased proportion (24%) had triple negative phenotype (Additional file 1: Table S2). Further studies in larger cohorts will be necessary to determine whether the triple negative phenotype is overrepresented in *PALB2* carriers with specific (founder) mutations or specific clinical presentations (early onset or bilateral disease).

Pathogenic or likely pathogenic *PALB2* mutations were observed in 2 of 89 families (2.2%) with 3 or more cases of breast cancer (Table 3A). Although not statistically significant, these numbers suggest a greater than 1% chance of identifying mutations in *PALB2* in families with 3 or more breast cancer cases where *BRCA1/2* mutations have been excluded.

**Table 3:** Total patient population screened

**(A)Number of Breast Cancer Cases in Families**							
	**Total**	**5 BC in family**	**4 BC in family**	**3 BC in family**	**2 BC in family**	**1 BC in family**	**0 BC in family**
**Female Unilateral Breast**	126	19	25	31	39	12	0
**Female Multiple Primary Breast**	24	1	4	2	12	5	0
**Male Breast**	12	0	0	1	4	7	0
**Breast and Ovarian**	4	1	1	0	1	1	0
**Ovarian**	9	0	2	2	2	1	2
**Total screened**	175	21	32	36	58	26	2
***PALB2*** mutations*	2	0	0	2	0	0	0
**B)Proximity to Breast Cancer Cases in Families**							
		**Total**	**FDR with BC**	**SDR with BC**	**TDR with BC**	**No relatives with BC**	
**Female Unilateral Breast**		126	79	28	7	12	
**Female Multiple Primary Breast**		24	11	5	3	5	
**Male Breast**		12	2	1	2	7	
**Breast and Ovarian**		4	2	0	1	1	
**Ovarian**		9	4	2	1	2	
**Total screened**		175	98	36	14	27	
***PALB2*** mutations*		2	1	0	1	0	

*Only the two definitely deleterious mutations were included in these tables. BC = breast cancer; FDR = first degree relative; SDR = second degree relative; TDR = third degree relative.

The majority of probands with a pathogenic or suspected pathogenic *PALB2* mutation had a first or second-degree relative with breast cancer (Table 3B), and if they did not, had a first degree or second-degree relative with a tumor suspected to be associated with a *BRCA2* or *PALB2* mutation (Additional file 1: Figure S2). Thus it does not appear to be worthwhile to screen families for *PALB2* mutations if the proband does not have close relatives who are affected by breast cancer or a *BRCA2/PALB2* related cancer.

Similar to the observations of Casadei *et al.*[13], the cancer profiles of the two *PALB2* mutation families seemed similar to cancer profiles of *BRCA2* families; including relatives affected with pancreatic cancer and melanoma. The frequencies of these cancers in the family histories of our cohort are described in Additional file 1: Table S5A and Additional file 1: Table S5B, respectively. Multiple studies have identified *PALB2* mutations in familial pancreatic cancer probands, several of which were found to have family histories including cases of breast cancer [8,29-31]. It should be noted that these mutations occur at relatively low frequencies (0–4%), which depend on the population being studied [8,32].

Whether or not *PALB2* mutations are associated with an increased risk of melanoma remains unclear and further studies are needed. Rahman *et al.*[5] described a proband with a truncating *PALB2* mutation (p.Y1183X) and diagnoses of melanoma at 47 years of age and breast cancer at 56 years. To the best of our knowledge, this is the only case in the literature describing melanoma in *PALB2* mutation carriers. In addition, Sabbaghian *et al.*[33] did not find a single *PALB2* mutation in 53 cases of familial melanoma. While we do not know *PALB2* status in the niece of our c.3507_3508delTC proband (given her young age she was not yet offered testing), the extremely young age of malignant melanoma diagnosis (at 11 years) is intriguing.

The contribution of *PALB2* mutations to ovarian cancer risk also remains uncertain with previous studies being conflicting (reviewed in 3,4). Some groups have observed an increased likelihood for *PALB2* mutation carriers to have relatives affected with ovarian cancer, albeit this was not a statistically significant finding [13]. One study which used massively parallel sequencing to examine the burden of loss of function mutations in tumor suppressor genes in women diagnosed with primary ovarian, peritoneal, or fallopian tube carcinoma identified 2 *PALB2* mutations in 360 probands tested [9]. It should be noted that the majority (139/175) of the families we screened did not contain any reported cases of ovarian cancer (Additional file 1: Table S5C).

Both the truncating pathogenic and the non-truncating frameshifting (and likely pathogenic) mutation in *PALB2* were identified amongst the 77 women diagnosed with premenopausal (<50 years old) unilateral breast cancer. Therefore, 2/77 (2.6%) mutations were identified in this particular group of women. Data from other reported series also suggests that *PALB2* mutations may be preferentially identified in premenopausal women, but not all studies report on the number of individuals in the series with diagnoses under the age of 50. Rahman *et al.*[5] noted that the median age of diagnosis for those with *PALB2* mutations in their study was 46 years, while those individuals without a *PALB2* mutation had a median age at diagnosis of 49 years. In their cohort, 8/10 probands with pathogenic mutations had their breast cancer diagnosed at or less than 50 years of age. In the case series reported by Southey *et al.*, 4/5 had breast cancer diagnosed at or less than 50 years of age [7]. In the study of cases with bilateral breast cancer reported by Tischkowitz [12], the median age at diagnosis of first breast cancer in the 5 women identified to carry a pathogenic mutation was 46 years. Notably in their series, none of the probands had an additional young onset case in the family. Taken together, these data suggest that it may be reasonable to preferentially offer *PALB2* clinical testing to *BRCA1/2-*negative breast and/or ovarian cancer families with multiple cases of breast cancer, including at least one case of premenopausal breast cancer.

Finally, although the sample size in our study is small, the presence of *PALB2* mutations in our cohort confirms that they are present at low frequency in familial breast cancer cases. It is also possible that deep intronic mutations may exist which would have escaped detection with our screening methodology.

## Conclusion

In 175 probands from a clinic based series of breast and/or ovarian cancer families with no detectable *BRCA1/BRCA2* mutations, we have identified two mutations in *PALB2*: one, c.3113G > A, is known to be deleterious and the other, c.3507_3508delTC (p.H1170Ffs*19), is likely to be deleterious. Both probands had family histories in which three or more individuals had been diagnosed with breast cancer. Both of these two families also had a family member with a diagnosis of another *BRCA2*-related cancer. *PALB2* mutations are not as frequent in the population as *BRCA1* and *BRCA2* mutations; however, the role of *PALB2* in breast cancer susceptibility is still significant, some studies suggesting that particular *PALB2* mutations may predispose individuals to breast cancer to a similar extent as *BRCA2*. It therefore warrants knowing who should be tested.

This study supports the notion that *PALB2* mutations are present at a relatively low frequency in hereditary breast cancer cases and that in women with breast cancer, who have a family history including 3 or more cases of breast cancer, *PALB2* testing may identify a mutation in >2% of cases and restricting testing to affected premenopausal women in such families may capture the vast majority of these mutations. Clinical testing could also be considered in the context of another *BRCA2*-related malignancy, and when at least one family member is diagnosed with premenopausal breast cancer.

## Competing interests

The authors declare that they have no competing interests.

## Authors’ contributions

All authors made substantial contributions to the acquisition and interpretation of data and critical revision of the manuscript. MT, ET and WDF made substantial contributions to the conception and design of the study. TH, RSS, ES, VH, NH and ET were involved in patient care/coordination of the study. TH, NH, ET, WDF wrote the manuscript. LC, NS, PP performed the molecular genetic studies. OA provided pathology expertise for the study. NH and LC performed the LOH studies on tumor tissue. All authors read and approved the final manuscript.

## Supplementary Material

Additional file 1: Figure S1 Predicted effect of truncating mutations on PALB2 protein. **Figure S2.** Pedigrees of the *PALB2* missense mutation-carrier families. **Figure S3.** The c.3507_3508delTC (p.H1170Ffs*19) variant is expressed in lymphoblastoid cells from the patient. **Table S1.** Ontario guidelines for molecular analysis of *BRCA1* and *BRCA2*. **Table S2.** Breast Tumor Characteristics. **Table S3.** Primers. **Table S4.** Variants found (n = 176) 89 individuals had no variants and 86 had 1 or more variants. **Table S5.** Total patient population screened. (A) Number of Pancreatic Cancer Cases in Families. (B) Number of Melanoma Cancer Cases in Families. (C) Number of Ovarian Cancer Cases in Families. Click here for file
