# Supplementary material for: Mutation analysis of PALB2 in BRCA1 and BRCA2-negative breast and/or ovarian cancer families from Eastern Ontario, Canada
Source: Hered Cancer Clin Pract. 2014 Aug 28;12(1):19. doi: 10.1186/1897-4287-12-19 (PMC4163678; doi:10.1186/1897-4287-12-19)
Supplement: Additional file 1: Figure S1 — Predicted effect of truncating mutations on PALB2 protein. Figure S2. Pedigrees of the PALB2 missense mutation-carrier families. Figure S3. The c.3507_3508delTC (p.H1170Ffs*19) variant is expressed in lymphoblastoid cells from the patient. Table S1. Ontario guidelines for molecular analysis of BRCA1 and BRCA2. Table S2. Breast Tumor Characteristics. Table S3. Primers. Table S4. Variants found (n = 176) 89 individuals had no variants and 86 had 1 or more variants. Table S5. Total patient population screened. (A) Number of Pancreatic Cancer Cases in Families. (B) Number of Melanoma Cancer Cases in Families. (C) Number of Ovarian Cancer Cases in Families. [file 1897-4287-12-19-S1.docx]

**Supplementary Materials**

**Figure S1. Predicted effect of truncating mutations on PALB2 protein.**


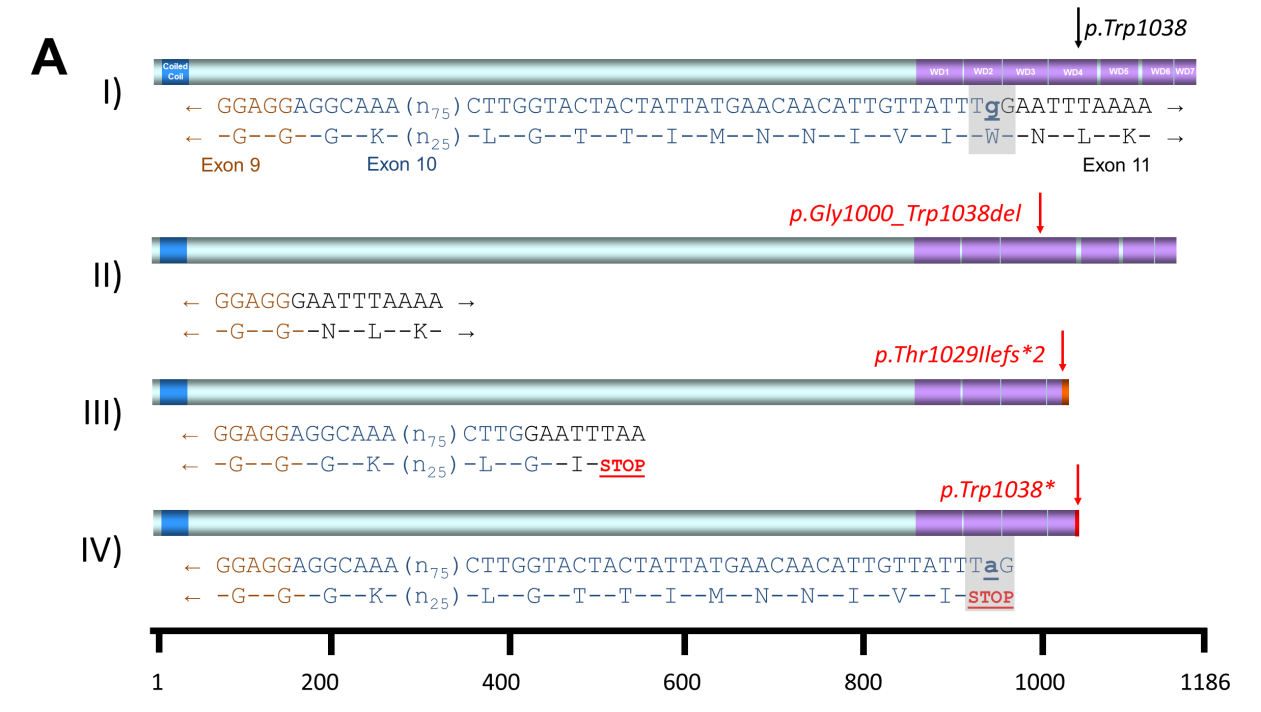
**(A)** Graphic representation of I) wild-type PALB2 and the three predicted mutated proteins resulting from the c.3113G>A mutation as described in Casadei et al, 2011 (Cancer Res 71(6):2222-9): II) skipping of the entire exon 10, in-frame, 56% of transcripts; III) out-of-frame 31 bp deletion from aberrant splicing, 40% of transcripts; IV) stop from point mutation, 4% of transcript.


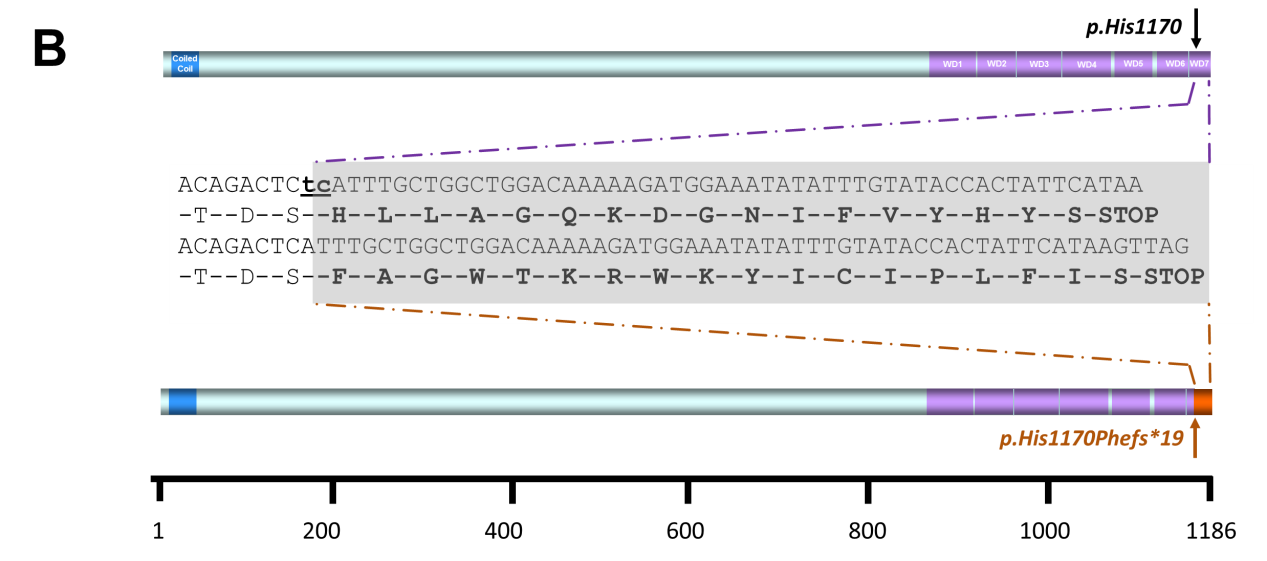
**(B)** Graphic representation of wild-type PALB2 (top) compared to the predicted mutated protein created by the c.3507_3508delTC mutation (bottom).

The axis denotes amino acid positions for both panels.


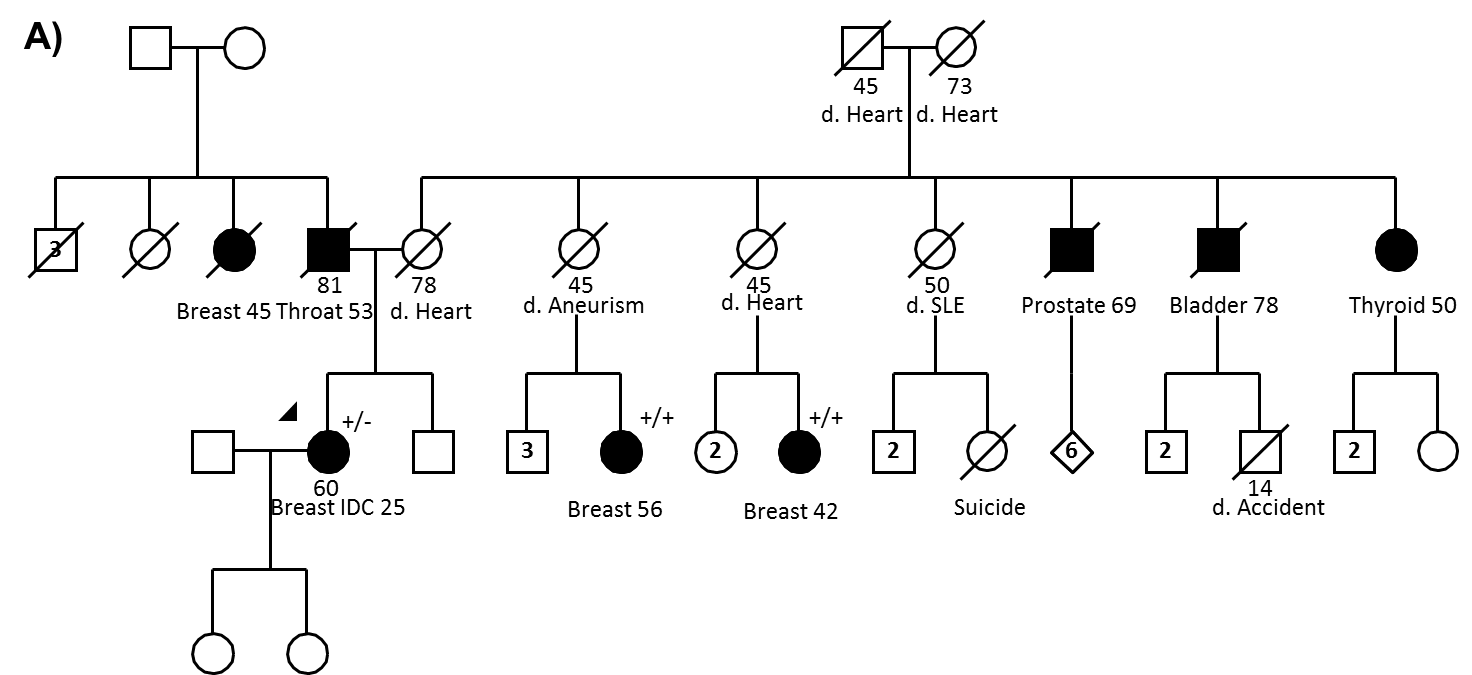
**Figure S2.** **Pedigrees of the *PALB2* missense mutation-carrier families**.

**(A)** Pedigree of the *PALB2* c.1846G>C, (p.D616H) mutation carrier family (French Canadian). **(B)** Pedigree of the *PALB2* c.3418T>G, (p.W1140G) mutation carrier family (British, French Canadian). **(C)** Pedigree of the *PALB2* c.3278A>G, (p.N1096S) mutation carrier family (Mainland Northern Portuguese). Probands are indicated by arrowheads. Individuals diagnosed with cancer are indicated with black filled symbols. All primary cancer diagnoses are indicated for each individual. Numbers within symbols represent multiple individuals. +/- = *PALB2* mutation identified; +/+ = *PALB2* mutation not present. SLE = systemic lupus erythematosis. IDC = invasive ductal carcinoma.


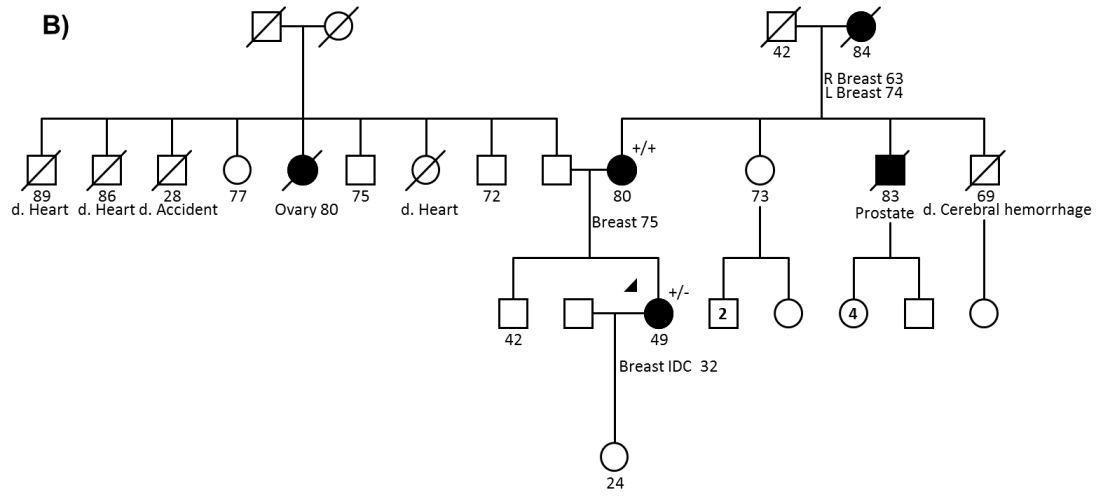

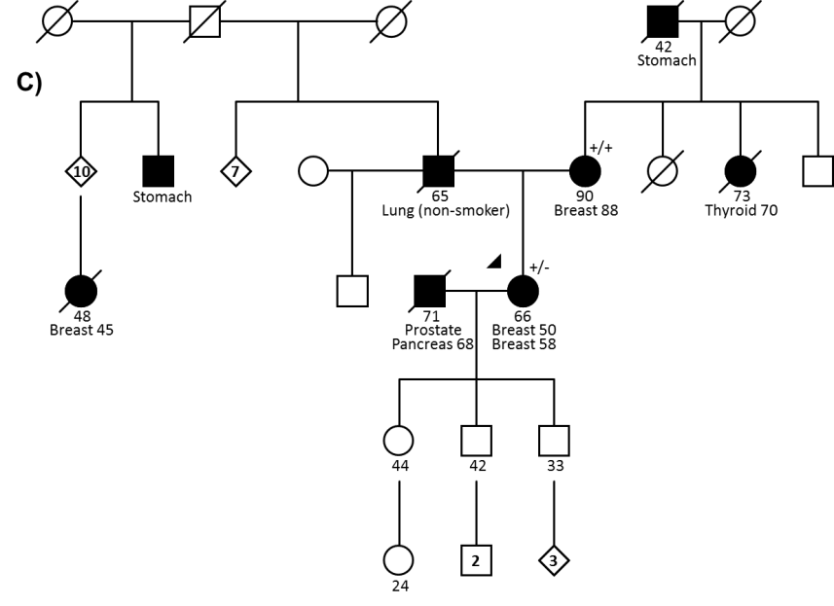


**Figure S3. The c.3507_3508delTC (p.H1170Ffs*19) variant is expressed in lymphoblastoid cells from the patient**


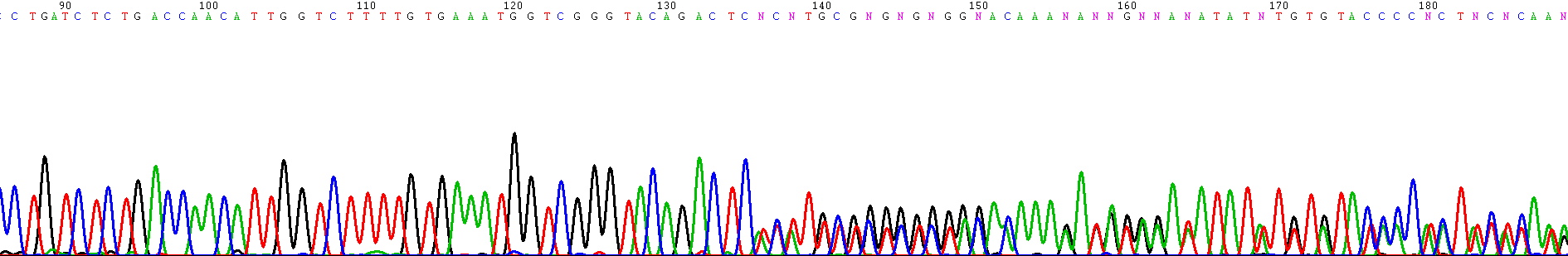


Chromatogram of cDNA derived from RNA of lymphoblastoid cells (LCLs). The arrow points to the site of the deletion. The heterozygous sequence is evidence that both alleles are expressed and that the mutant allele containing the deletion is not subject to nonsense-mediated decay.

**Table S1.**  Ontario guidelines for molecular analysis of BRCA1 and BRCA2

| **Cohort 1** | *At least one case of cancer:* | 1. Ashkenazi Jewish and breast cancer <50 years, or ovarian cancer at any age (limited to AJ mutations) 2. Breast cancer <35 years 3. Male breast cancer 4. Invasive serous ovarian cancer at any age |
| --- | --- | --- |
|  | *At least two cases of cancer on the same side of the family:* | 1. Breast cancer <60years, and a first or second degree relative with ovarian cancer or male breast cancer 2. Breast and ovarian in the same individual or bilateral breast cancer with the first case <50years 3. Two cases of breast cancer, both <50 years, in first or second-degree relatives 4. Two cases of ovarian cancer, any age, in first of second-degree relatives 5. Ashkenazi Jewish and breast cancer at any age and any family history of breast or ovarian cancer (limited to AJ mutations) |
|  | *At least 3 cases of cancer on the same side of the family:* | 1. Three or more cases of breast or ovarian cancer at any age |
| **Cohort 2** | *At least one case of cancer:* | 1. Ashkenazi Jewish and breast cancer <50 years 2. Breast cancer <35 years 3. Male breast cancer |
|  | *At least two cases of cancer on the same side of the family:* | 1. Breast cancer <60years, and a first or second degree relative with male breast cancer 2. Bilateral breast cancer with the first case <50years 3. Two cases of breast cancer, both <50 years, in first or second-degree relatives |
|  | *At least 3 cases of cancer on the same side of the family:* | 1. Three or more cases of breast cancer, at least one diagnosis premenopausal |

**Table S2.** Breast Tumor Characteristics

|  | **Female Unilateral** | | **Female Multiple Primary** | | **Male Breast** | | **Breast and Ovarian** | |
| --- | --- | --- | --- | --- | --- | --- | --- | --- |
| ***Immunohistochemistry*** | N | % | N | % | N | % | N | % |
| ***Estrogen Receptor:*** |  |  |  |  |  |  |  |  |
| Number of tumours assessed | 109 |  | 29 |  | 12 |  | 4 |  |
| Positive | 89/109 | 81.7 | 21/29 | 72.4 | 12/12 | 100 | 4/4 | 100 |
| Negative | 19/109 | 17.4 | 8/29 | 27.6 | 0/12 | 0 | 0/4 | 0 |
| Borderline | 1/109 | 0.9 | 0/29 | 0 | 0/12 | 0 | 0/4 | 0 |
| ***Progesterone Receptor:*** |  |  |  |  |  |  |  |  |
| Number of tumours assessed | 108 |  | 27 |  | 12 |  | 4 |  |
| Positive | 79/108 | 73.1 | 15/27 | 55.6 | 12/12 | 100 | 3/4 | 75.0 |
| Negative | 26/108 | 24.1 | 11/27 | 40.7 | 0/12 | 0 | 1/4 | 25.0 |
| Borderline | 3/108 | 2.8 | 1/27 | 3.7 | 0/12 | 0 | 0/4 | 0 |
| ***Her2 Overexpression*** |  |  |  |  |  |  |  |  |
| Number of tumours assessed | 85 |  | 17 |  | 8 |  | 3 |  |
| Positive | 20/85 | 23.5 | 2/17 | 11.7 | 0/8 | 0 | 1/3 | 33.3 |
| Negative | 62/85 | 73.0 | 15/17 | 88.2 | 8/8 | 100 | 2/3 | 66.7 |
| Borderline | 3/85 | 3.5 | 0/17 | 0 | 0/8 | 0 | 0/3 | 0 |
| ***Number of tumours assessed for all three markers*** | 85 |  | 17 |  | 8 |  | 3 |  |
| ***Triple Negative*** | 9/85 | 10.6 | 4/17 | 23.5 | 0/8 | 0 | 0/3 | 0 |

**Table S3**. Primers

| **Primer Name** | **Primer Sequence** |
| --- | --- |
| PALB2_3507.175F | 5’-TCCAGAAAATTGTGTTTTCACTTT-3’ |
| PALB2_3507.175R | 5’-GGGACTTACTTCTCGGTCAGTGT-3’ |
| PALB2_W1140G_162F | 5’-TCTTCTTTGTATGCTATCAGGTTCC-3’ |
| PALB2_W1140G_162R | 5’-CCATTTCACAAAAGACCAATGTT-3’ |
| PALB2_W1038x_176F | 5’-GCCCCCTGAGGAGACTATACTAA-3’ |
| PALB2_W1038x_176R | 5’-AATCTTCACAACAACCCTGTAAAA-3’ |

**Table S4.** Variants found (n=176) **89 individuals had no variants and 86 had 1 or more variants**

| **Known benign SNPs** | **number of cases** | **Likely non-pathogenic** | **number of cases** | **Previously unreported** | **number of cases** | **Likely Pathogenic** | **number of cases** | **Known Pathogenic** | **number of cases** |
| --- | --- | --- | --- | --- | --- | --- | --- | --- | --- |
| -47G>A rs8053188 | 1  1^†††^  7^**^ | c.298C>T p.L100F rs61756147 | 1 | c.111+11C>T | 1^*^ | c.1846G>C p.D616H | 1^§§^ | c.3113G>A p.W1038* | 1^*^ |
| c.212-58A>C rs80291632 | 1^†^  8^§§^ | c.1470 C>T p.P490P rs45612837 | 1^††^ | c.344G>T p.G115V | 1 | c.3418T>G p.W1140G rs62625283 | 1 | c.3507_3508delTC p.H1170Ffs*19 | 1 |
| c.1010 T>C p.L337S rs45494092 | 4  1^††^ | c.1676 A>G p.Q559R rs152451 | 1  12^§^  8^§§^  1^†^ |  |  | c.3287A>G p.N1096S | 1 |  |  |
| c.2329G>A p.D777N rs148026749 | 1 | c.2014 G>C p.E672Q rs45532440 | 1^†^  8^§§^ |  |  |  |  |  |  |
| c.2586+58C>T rs249954 | 43  8^**^  12^§^  8^§§^  1^†^  1^††^ | c.2590 C>T p.P864S rs45568339 | 1^†††^ |  |  |  |  |  |  |
| c.2993 G>A p.G998E rs45551636 | 8^§§^ | c.629C>T p.P210L rs57605939 | 1^**^ |  |  |  |  |  |  |
| c.3300 T>G p.T1100T rs45516100 | 1^†^  8^§§^ |  |  |  |  |  |  |  |  |

^*^ same patient; ^**^ 7 cases have both SNPs and 1 case has an additional SNP; ^***^ 1 case included in ^§§^; ^§^ 12 cases have both SNPs; ^§§^ 8 cases have 6 SNPs and one case has an additional SNP; ^†^ 1 case has 5 SNPs;^††^ 1 case has 3 SNPs;^†††^ 1 case has both SNPs

**Table S5.** Total patient population screened

**(A) Number of Pancreatic Cancer Cases in Families**

|  | **Total** | **3 PaC in family** | **2 PaC in family** | **1 PaC in family** | **0 PaC in family** |
| --- | --- | --- | --- | --- | --- |
| **Female Unilateral Breast** | 126 | 0 | 1 | 13 | 112 |
| **Female Multiple Primary Breast** | 24 | 0 | 0 | 4 | 20 |
| **Male Breast** | 12 | 0 | 0 | 1 | 11 |
| **Breast and Ovarian** | 4 | 0 | 0 | 1 | 3 |
| **Ovarian** | 9 | 0 | 0 | 0 | 9 |
| **Total screened** | 175 | 0 | 1 | 19 | 155 |
| ***PALB2* mutations*** | 2 | 0 | 0 | 1 | 1 |

**(B) Number of Melanoma Cancer Cases in Families**

|  | **Total** | **3 ME in family** | **2 ME in family** | **1 ME in family** | **0 ME in family** |
| --- | --- | --- | --- | --- | --- |
| **Female Unilateral Breast** | 126 | 0 | 3 | 16 | 107 |
| **Female Multiple Primary Breast** | 24 | 0 | 0 | 2 | 22 |
| **Male Breast** | 12 | 0 | 0 | 1 | 11 |
| **Breast and Ovarian** | 4 | 0 | 0 | 1 | 3 |
| **Ovarian** | 9 | 0 | 0 | 0 | 9 |
| **Total screened** | 175 | 0 | 3 | 20 | 152 |
| ***PALB2* mutations*** | 2 | 0 | 0 | 1 | 1 |

**Table S5.** Total patient population screened (continued)

**(C) Number of Ovarian Cancer Cases in Families**

|  | **Total** | **4 OC in family** | **3 OC in family** | **2 OC in family** | **1 OC in family** | **0 OC in family** |
| --- | --- | --- | --- | --- | --- | --- |
| **Female Unilateral Breast** | 126 | 0 | 0 | 4 | 16 | 106 |
| **Female Multiple Primary Breast** | 24 | 0 | 0 | 1 | 4 | 19 |
| **Male Breast** | 12 | 0 | 0 | 0 | 0 | 12 |
| **Breast and Ovarian** | 4 | 0 | 0 | 0 | 2 | 2 |
| **Ovarian** | 9 | 0 | 0 | 3 | 6 | 0 |
| **Total screened** | 175 | 0 | 0 | 8 | 28 | 139 |
| ***PALB2* mutations*** | 2 | 0 | 0 | 0 | 0 | 2 |

*we only included the two definitely deleterious mutations in these tables. PaC = Pancreatic Cancer; Me = Melanoma; OC = Ovarian Cancer
